# Assessement of educational burnout among medical students

**DOI:** 10.1192/j.eurpsy.2025.2006

**Published:** 2025-08-26

**Authors:** I. Baati, N. Regaieg, H. Trigui, M. Ben Jemaa, F. Cherif, I. Feki, F. Guermazi, J. Masmoudi

**Affiliations:** 1 Psychiatry A Department; 2 Epidemiology department, University of Sfax, Faculty of medicine of Sfax, Sfax, Tunisia

## Abstract

**Introduction:**

Burnout is characterized by emotional exhaustion, depersonalization, and reduced personal accomplishment. Medical students are particularly susceptible to academic burnout compared to students in other disciplines, due to the demanding nature of their training, which offers limited time and resources.

**Objectives:**

Our study aimed to assess academic burnout in a sample of students from the Faculty of Medicine of Sfax, Tunisia, as well as the link between these two entities.

**Methods:**

This was a cross-sectional and descriptive study, conducted using GOOGLE FORMS in February and March 2024, with a sample of students from the Faculty of Medicine in Sfax, Tunisia.

Data collection was carried out based on a questionnaire including an information sheet and the Maslach Burnout Inventory-Student Survey (MBI-SS) assessing academic burnout according to a three-dimensional structure : Emotional Exhaustion (EE) due to the study requirements; Cynicism (CY), which refers to a detached attitude towards one’s studies and Academic Effectiveness (AE) linked to a sense of achievement as a student.

**Results:**

Our study included 154 students. They were predominantly females (64.9%). Their median age was 22 years (IQR = [20 – 23 years]).

According to the MBI-SS, the prevalence of academic burnout was 98.1%.

The distribution of scores showed that academic burnout was mild in 15.9% of cases, moderate in 43.7% of cases and severe in 40.4% of cases.

The median scores on the EE, CY and AE dimensions were 17 (IQR = [13 – 20]) ; 11.5 (IQR= [7 – 16]) and19 (IQR = [15 – 23]), respectively.

The distribution of medical students according to the level of academic burnout is illustrated in Figure 1.

**Conclusions:**

Our results showed that medical students experience high levels of educational burnout. To mitigate this high prevalence, it essentiel to raise awareness about its symptoms and to implement educational programs focused on mindfulness and well-being. Higher education institutions play a vital role in supporting students’ professional development and resilience by fostering a healthy learning environment and providing psychological and pedagogical support.

**Disclosure of Interest:**

None Declared

